# Supplementary material for: Large-scale spatial variable gene atlas for spatial transcriptomics
Source: ArXiv. 2026 Sep 20:arXiv:2510.07653v4. Preprint. [Version 4] (PMC13685593)
Supplement: Supplementary file 1 [file NIHPP2510.07653v4-supplement-1.pdf]

A Supplementary Figures

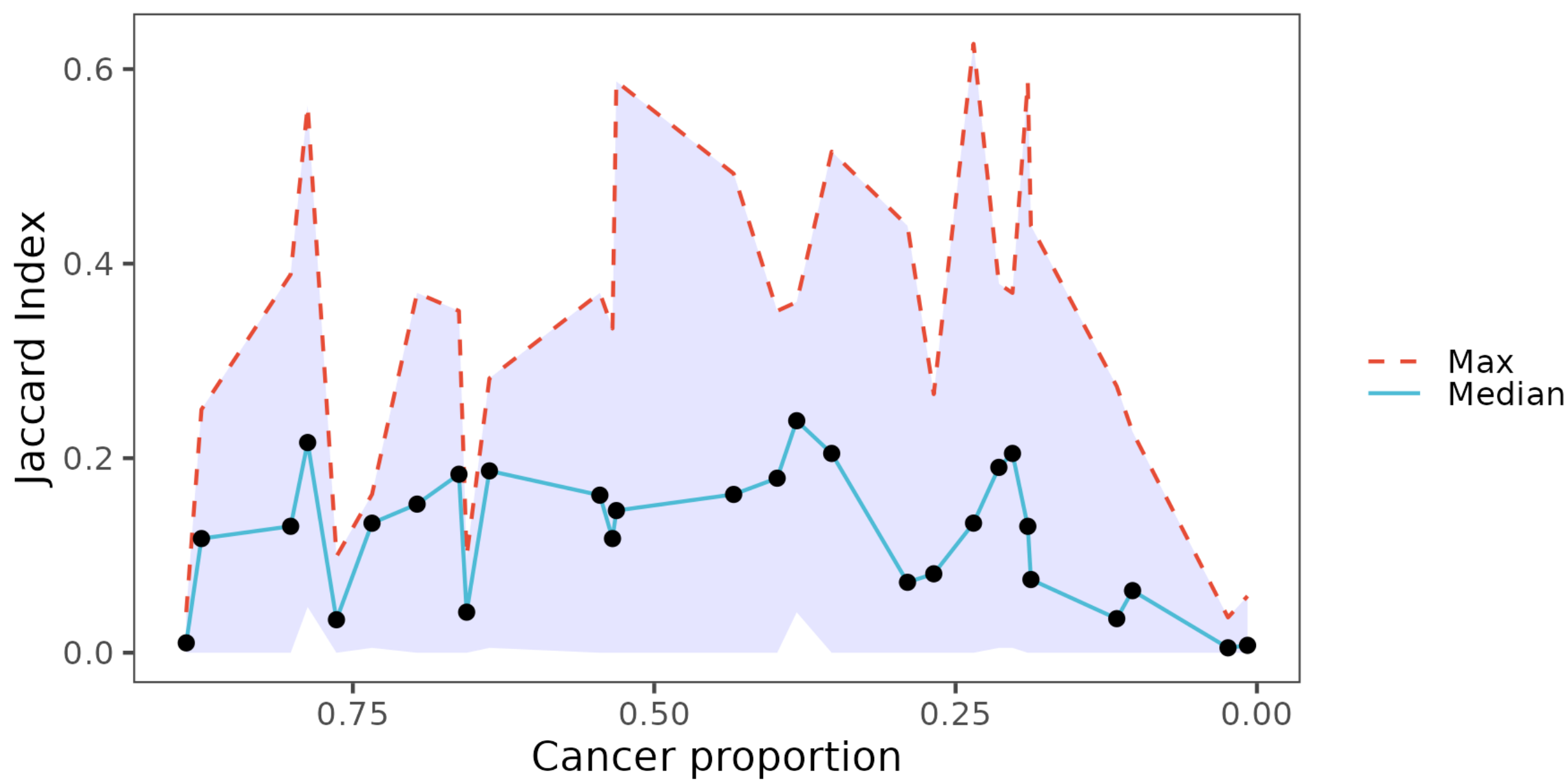

Supplementary Figure S1: Slide-level performance sorted by cancer proportion. Shaded area indicates the minimal and maximal Jaccard Index within methods, and the black dots indicate the median Jaccard Index.

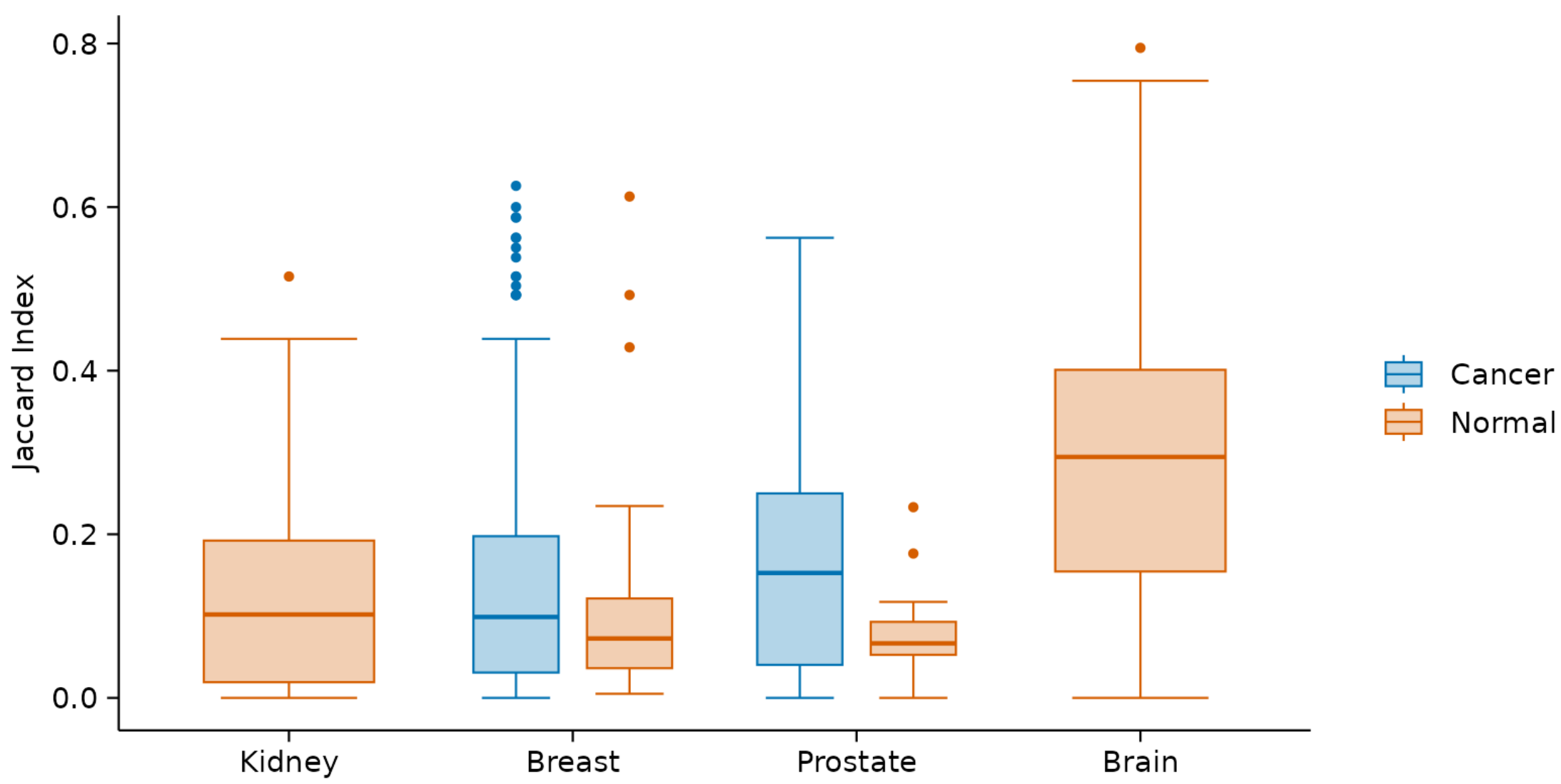

Supplementary Figure S2: Jaccard Index between SVG sets and DE gene sets across all methods, stratified by tissue and disease status.

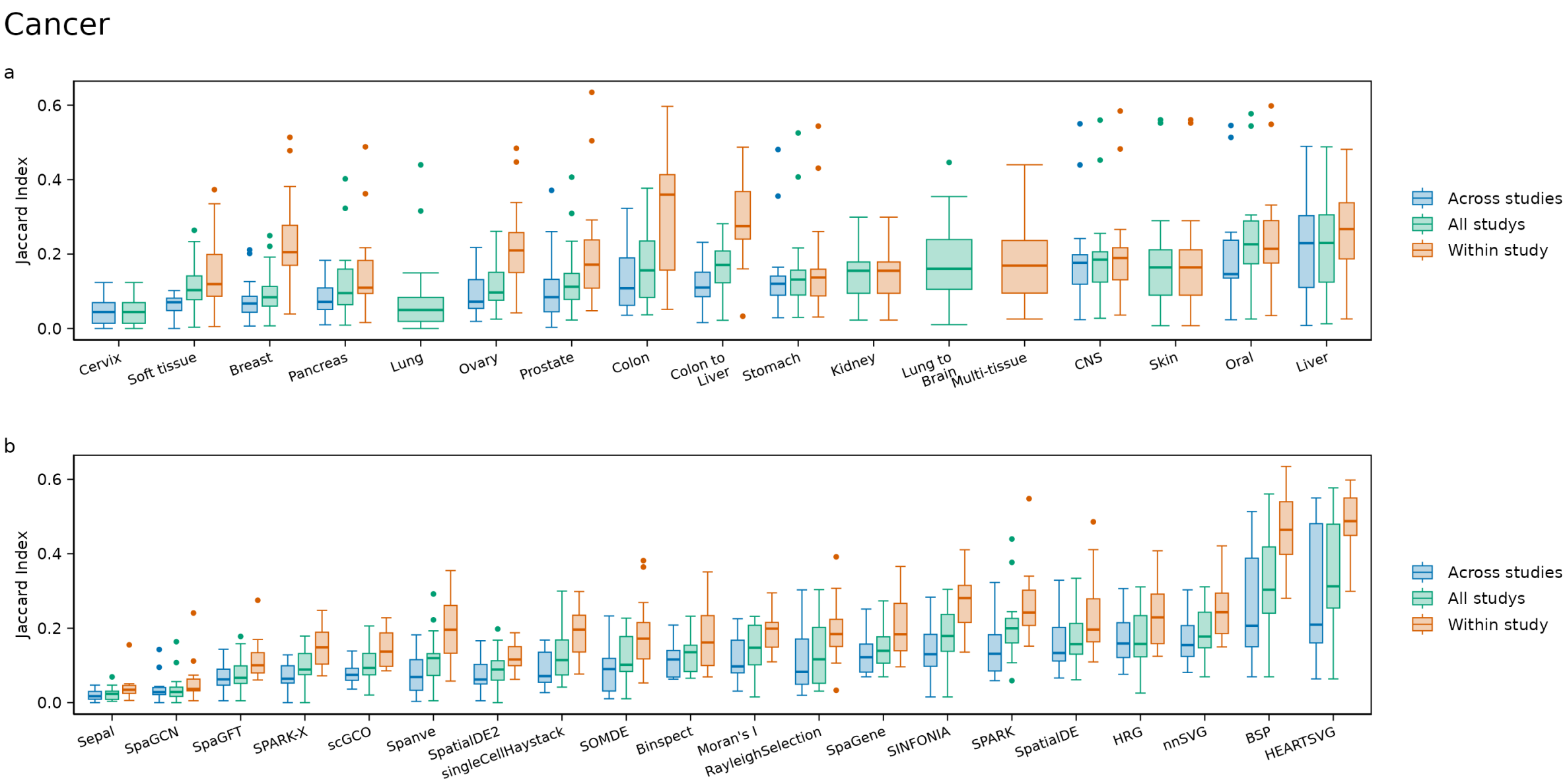

Supplementary Figure S3: Jaccard Index between SVG sets and DE gene sets on cancer slides, stratified by (a) tissue type and study types and (b) methods and study types.

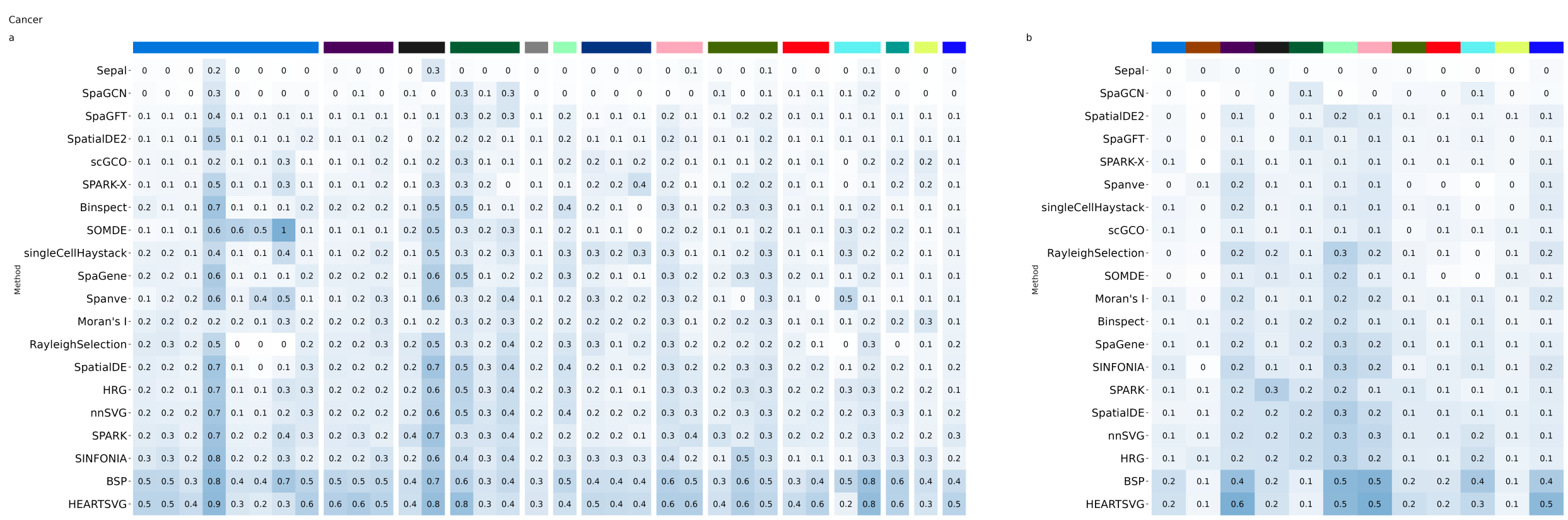

Supplementary Figure S4: Average Jaccard Index between SVG sets and DE gene sets on cancer slides of each method for (a) with-in study evaluation (b) across study evaluation.

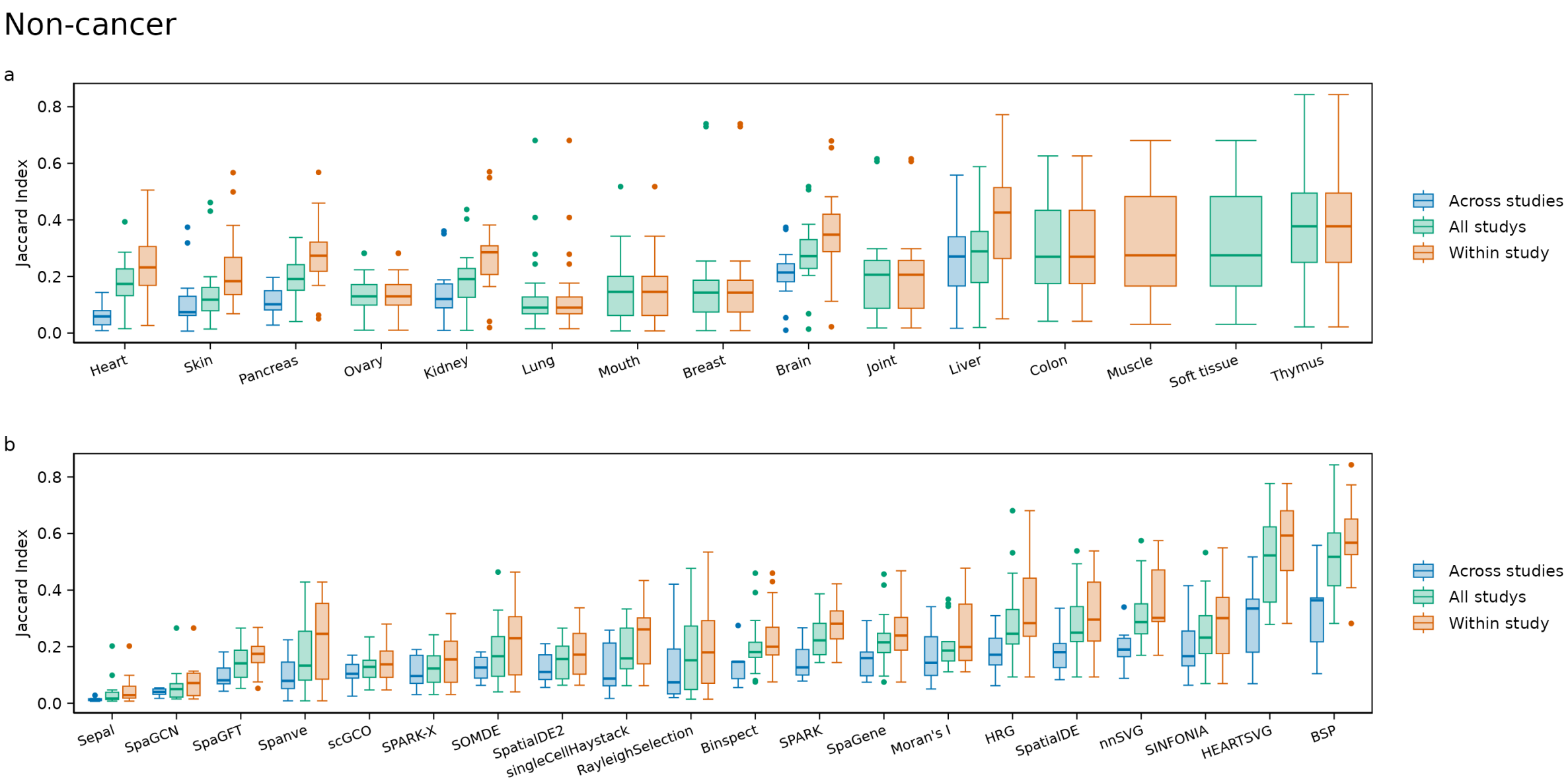

Supplementary Figure S5: Jaccard Index between SVG sets and DE gene sets on non-cancer slides, stratified by (a) tissue type and study types and (b) methods and study types.

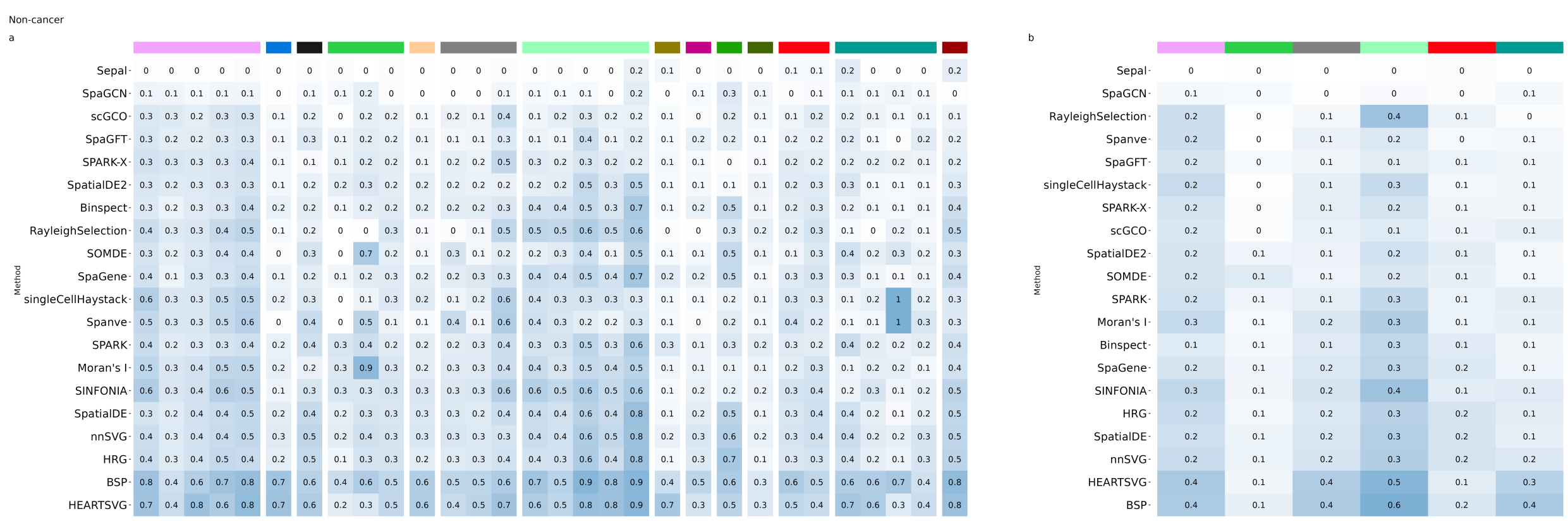

Supplementary Figure S6: Average Jaccard Index between SVG sets and DE gene sets on non-cancer slides of each method for (a) with-in study evaluation (b) across study evaluation.

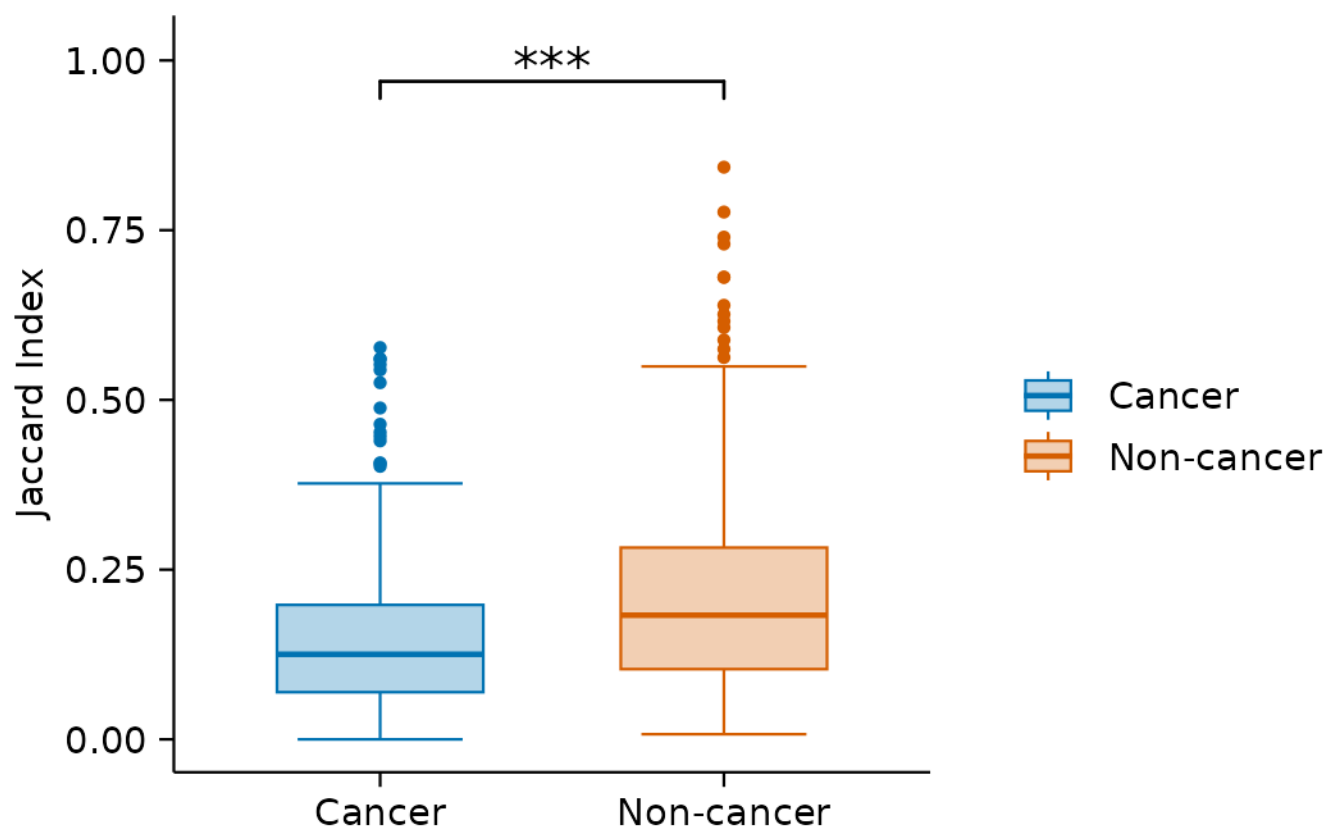

Supplementary Figure S7: Jaccard Index between SVG sets and DE gene sets, stratified by disease status.

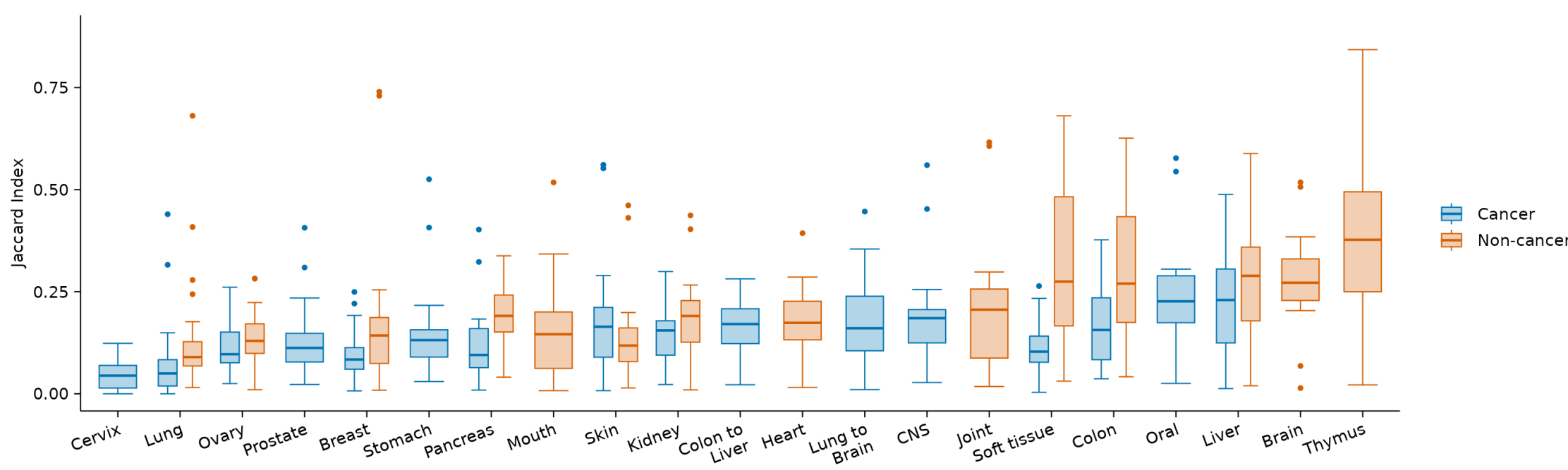

Supplementary Figure S8: Jaccard Index between SVG sets and DE gene sets, stratified by disease status and tissue types.

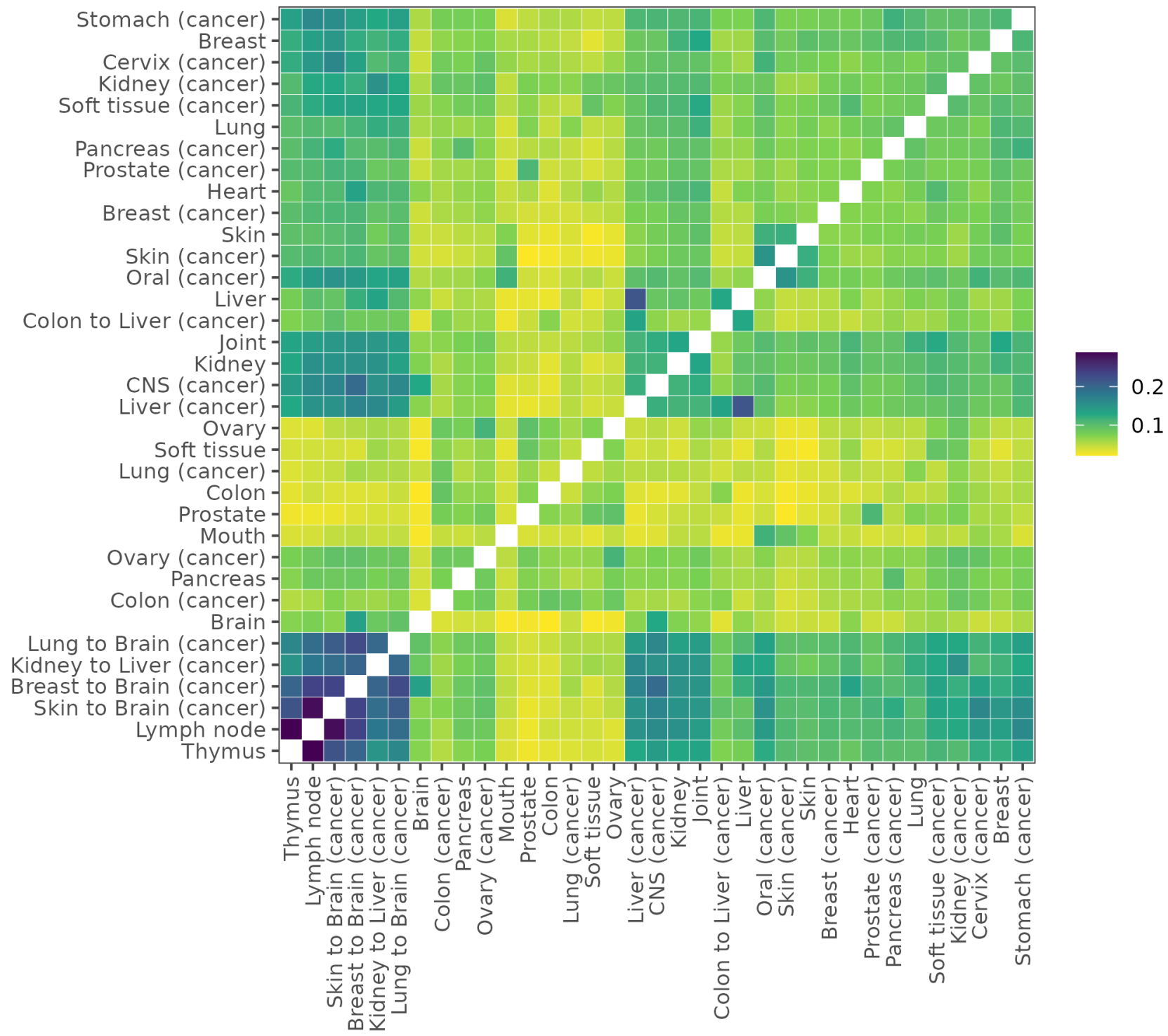

Supplementary Figure S9: Jaccard Index similarity between all tissue types

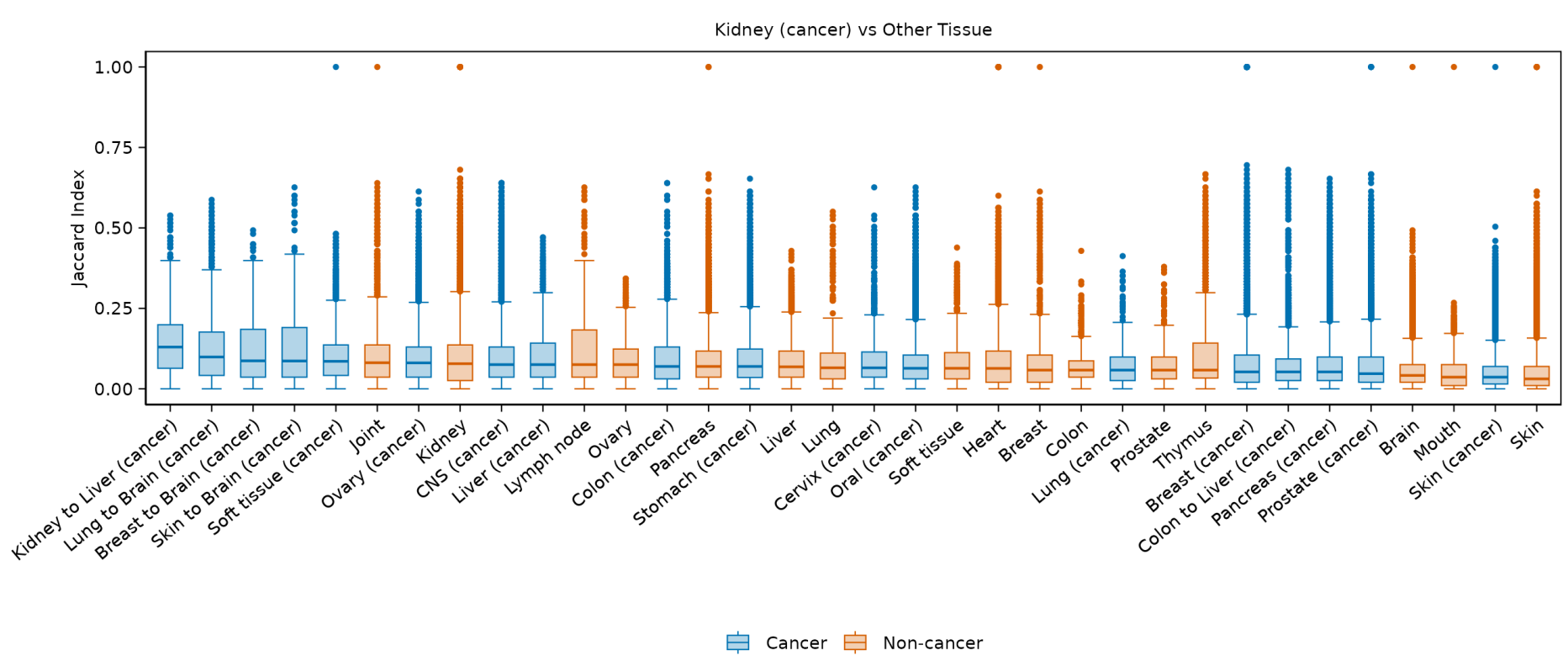

Supplementary Figure S10: Jaccard Index similarity between Kidney (cancer) vs Other Tissue

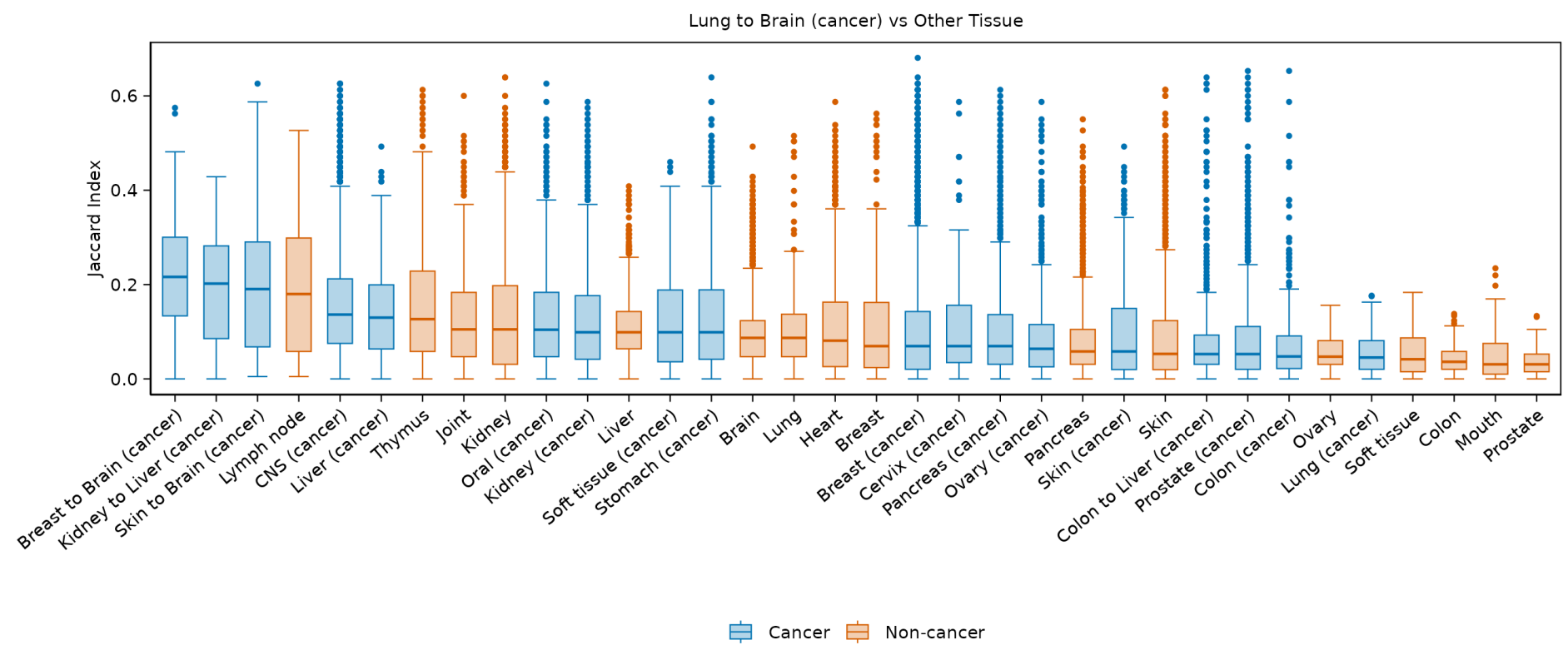

Supplementary Figure S11: Jaccard Index similarity between Lung to Brain (cancer) vs Other Tissue

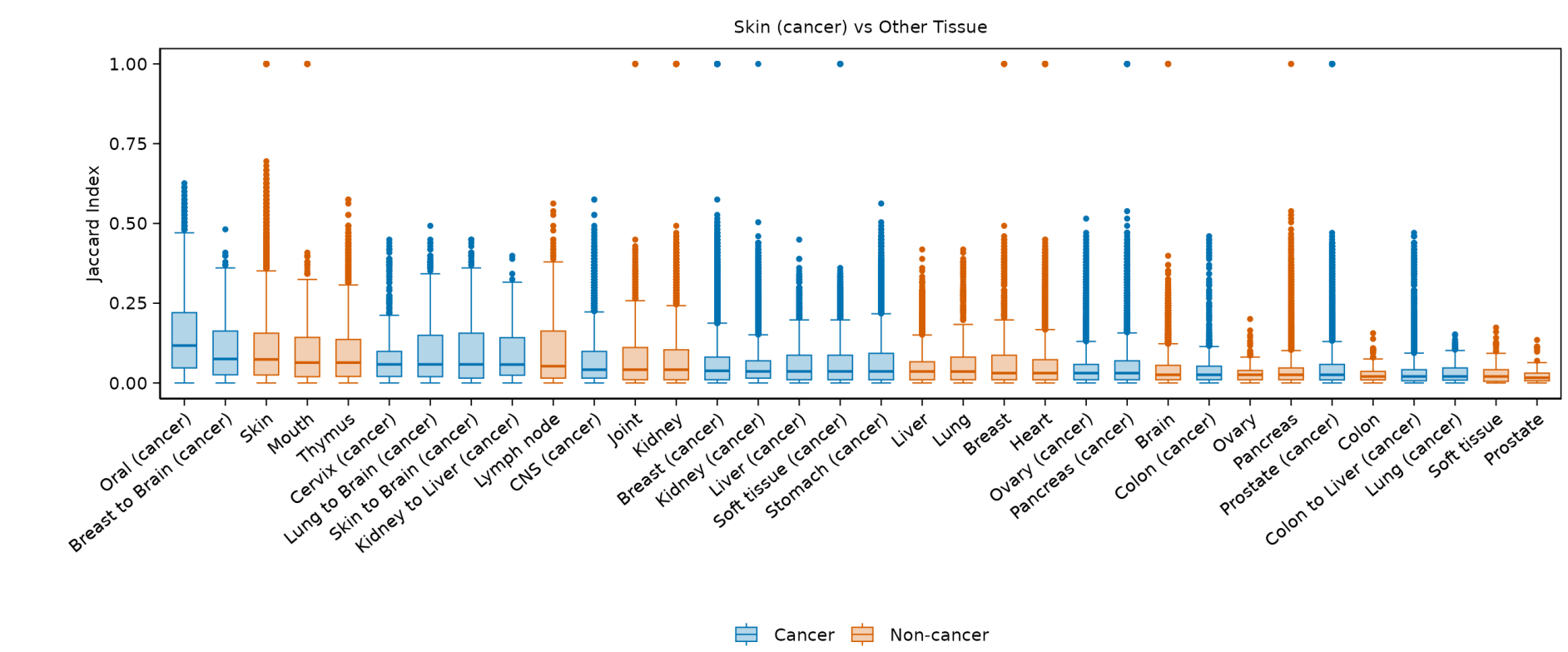

Supplementary Figure S12: Jaccard Index similarity between Skin (cancer) vs Other Tissue

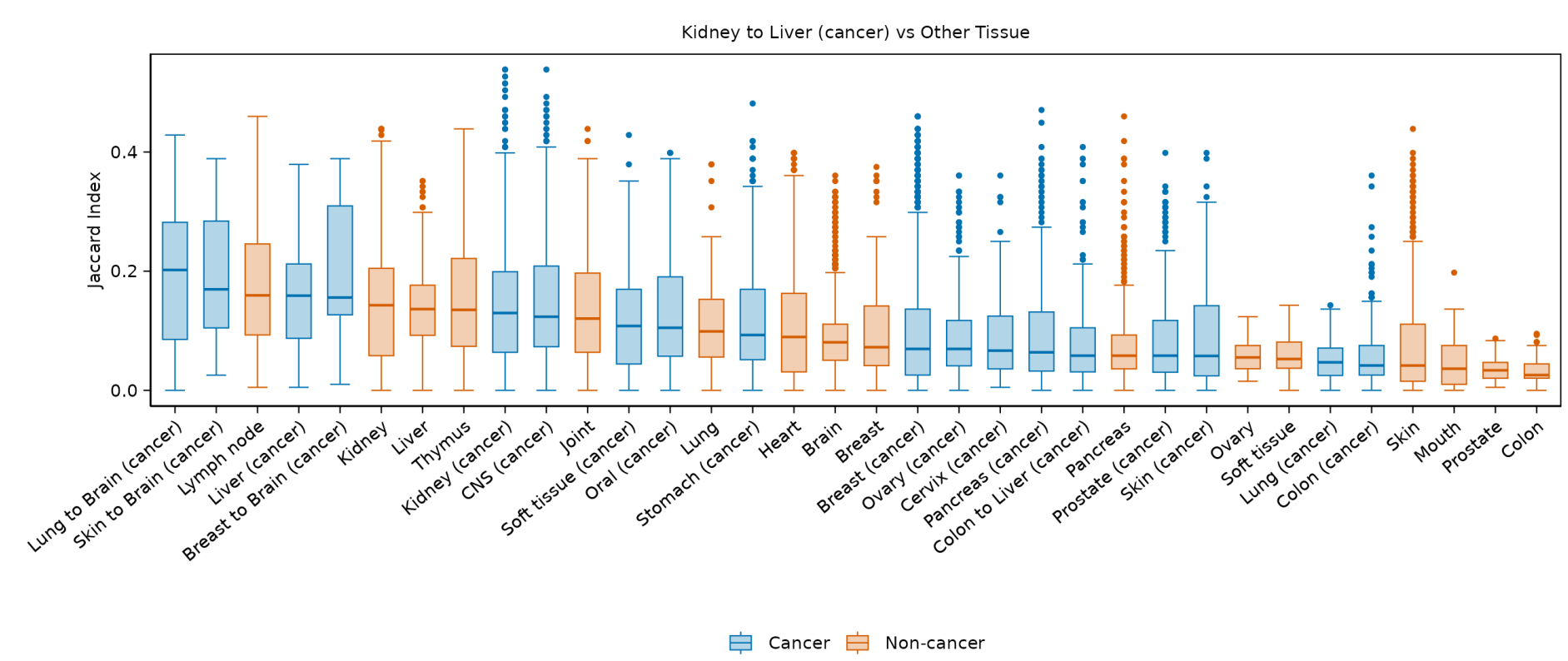

Supplementary Figure S13: Jaccard Index similarity between Kidney to Liver (cancer) vs Other Tissue

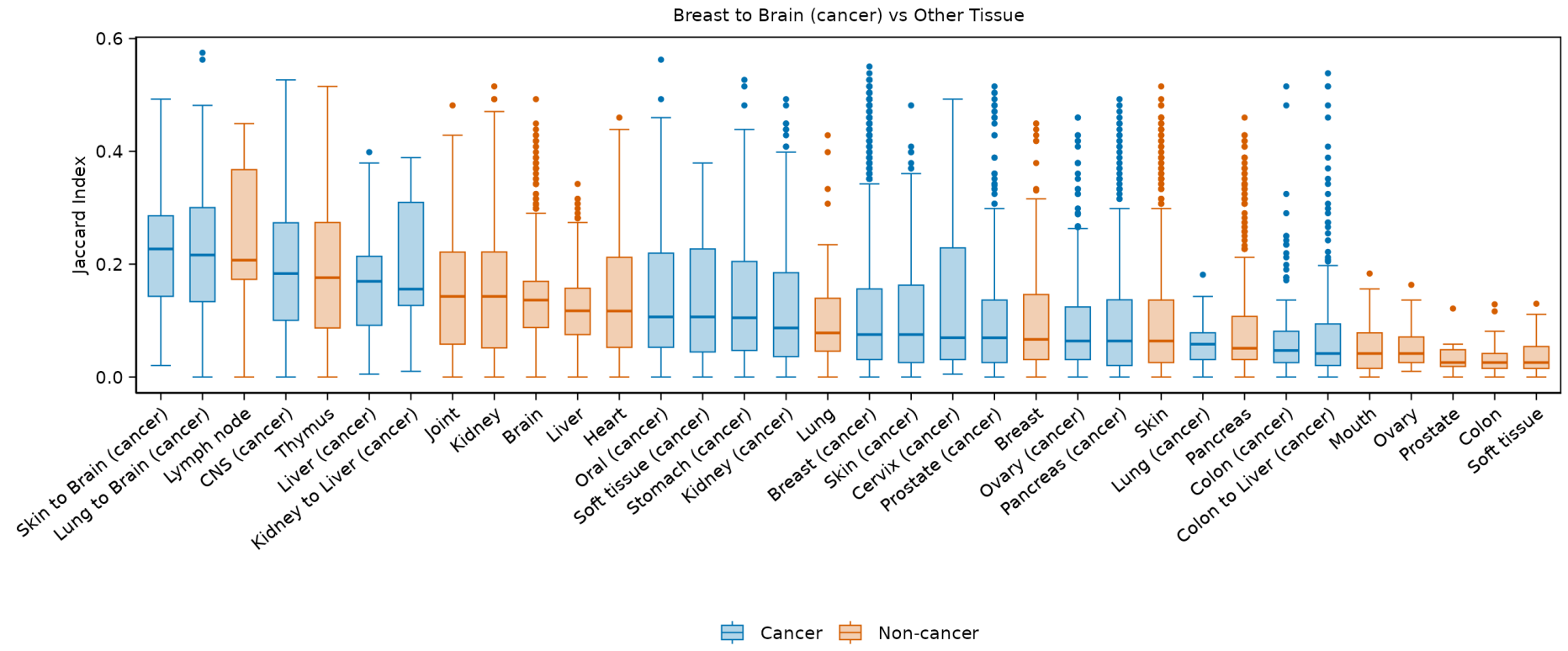

Supplementary Figure S14: Jaccard Index similarity between Breast to Brain (cancer) vs Other Tissue

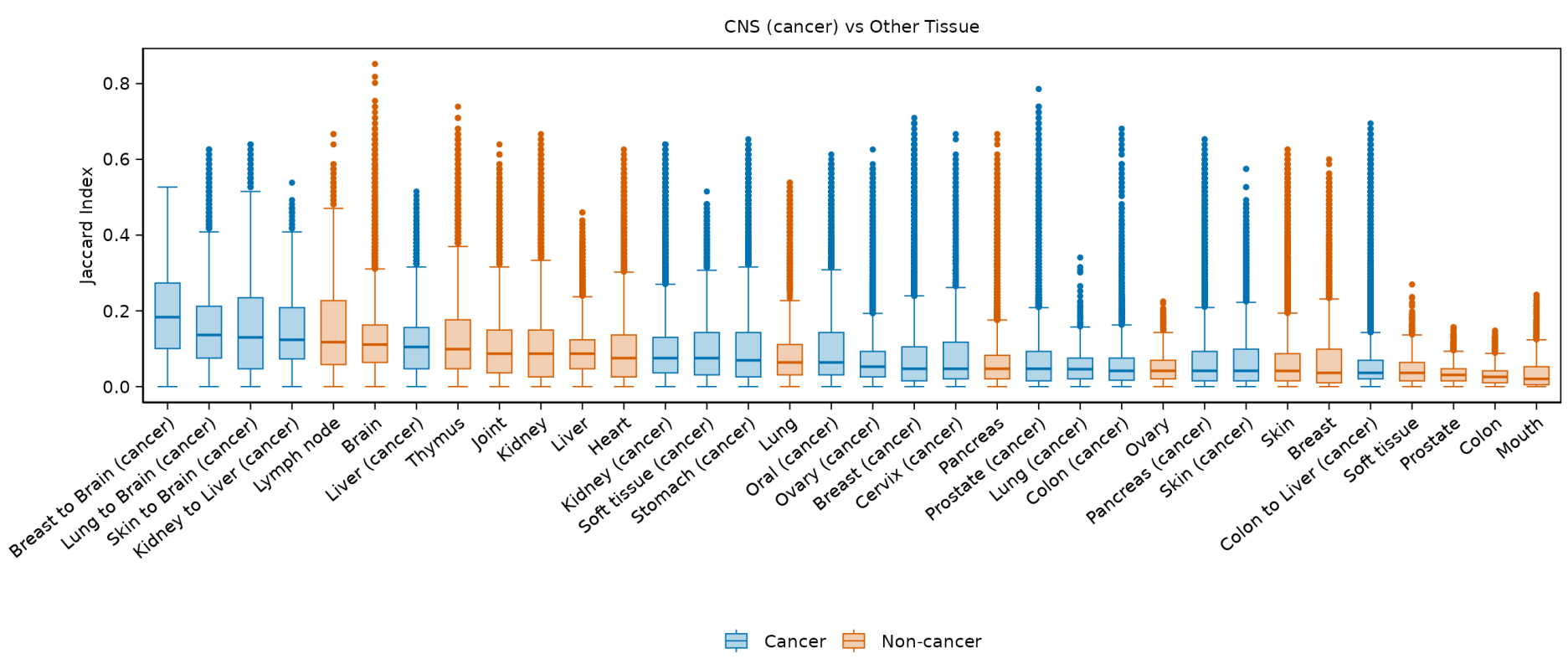

Supplementary Figure S15: Jaccard Index similarity between CNS (cancer) vs Other Tissue

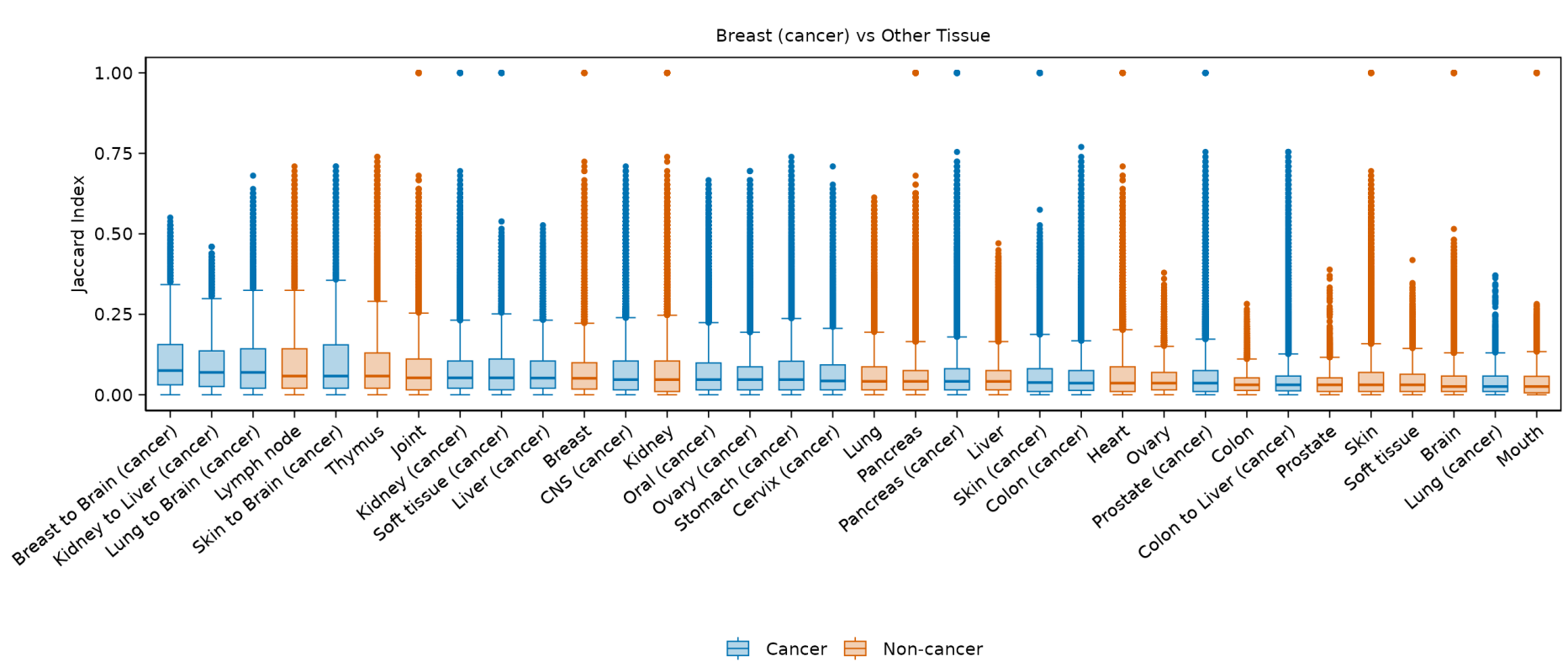

Supplementary Figure S16: Jaccard Index similarity between Breast (cancer) vs Other Tissue

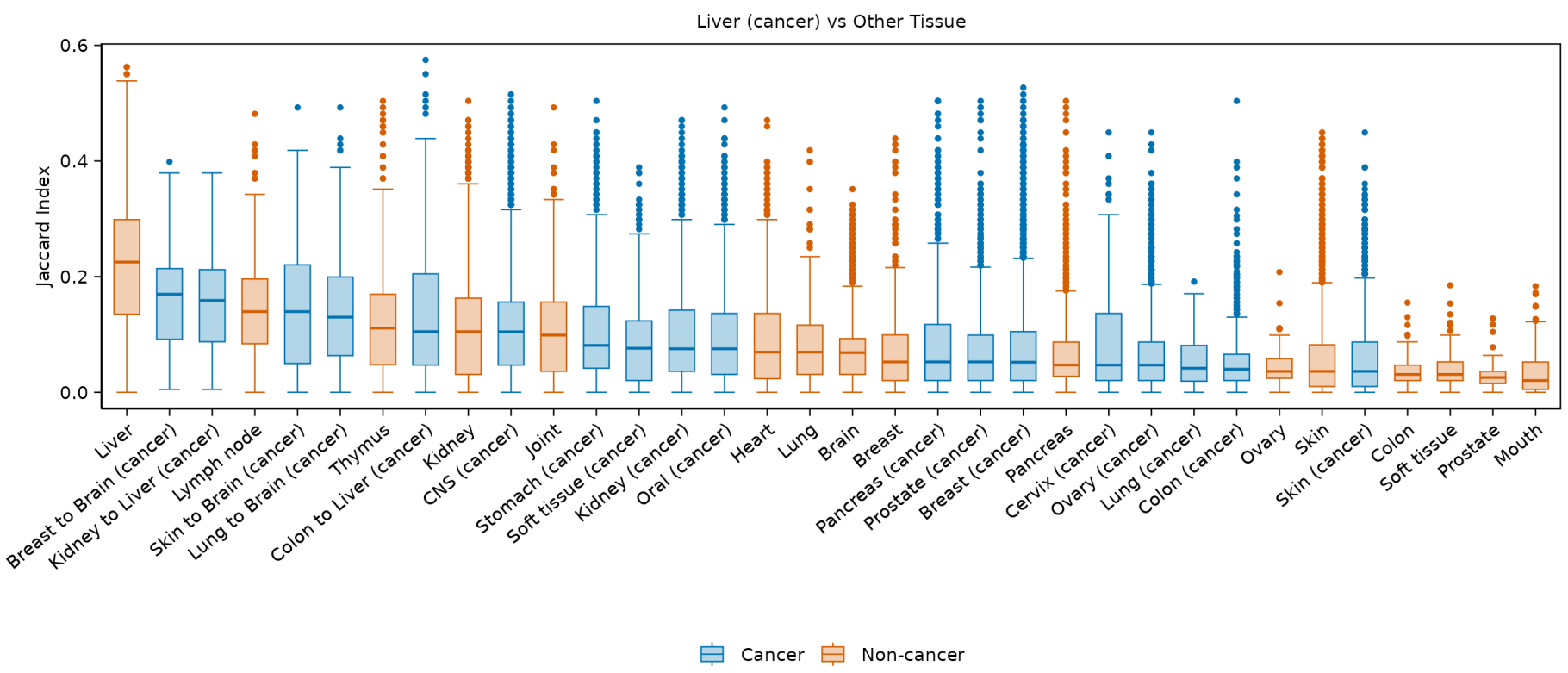

Supplementary Figure S17: Jaccard Index similarity between Liver (cancer) vs Other Tissue

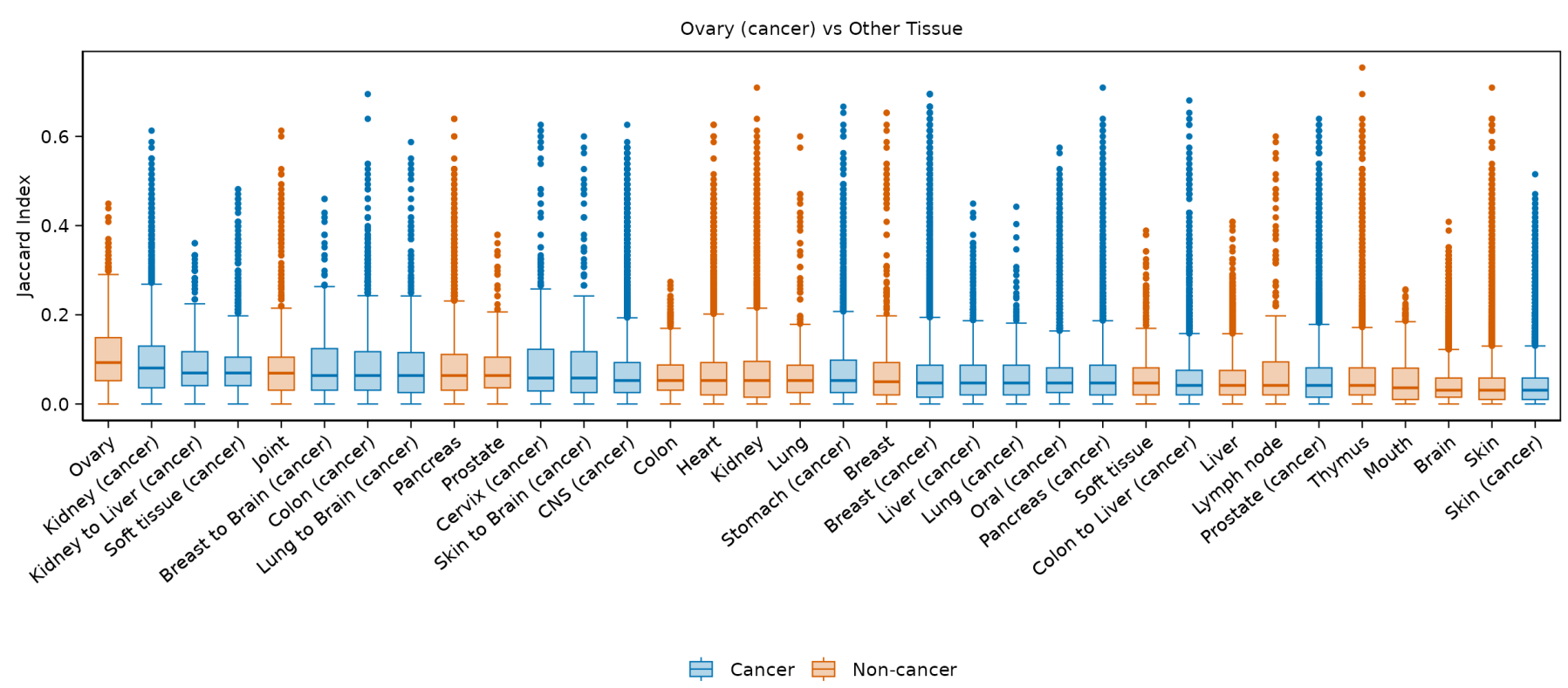

Supplementary Figure S18: Jaccard Index similarity between Ovary (cancer) vs Other Tissue

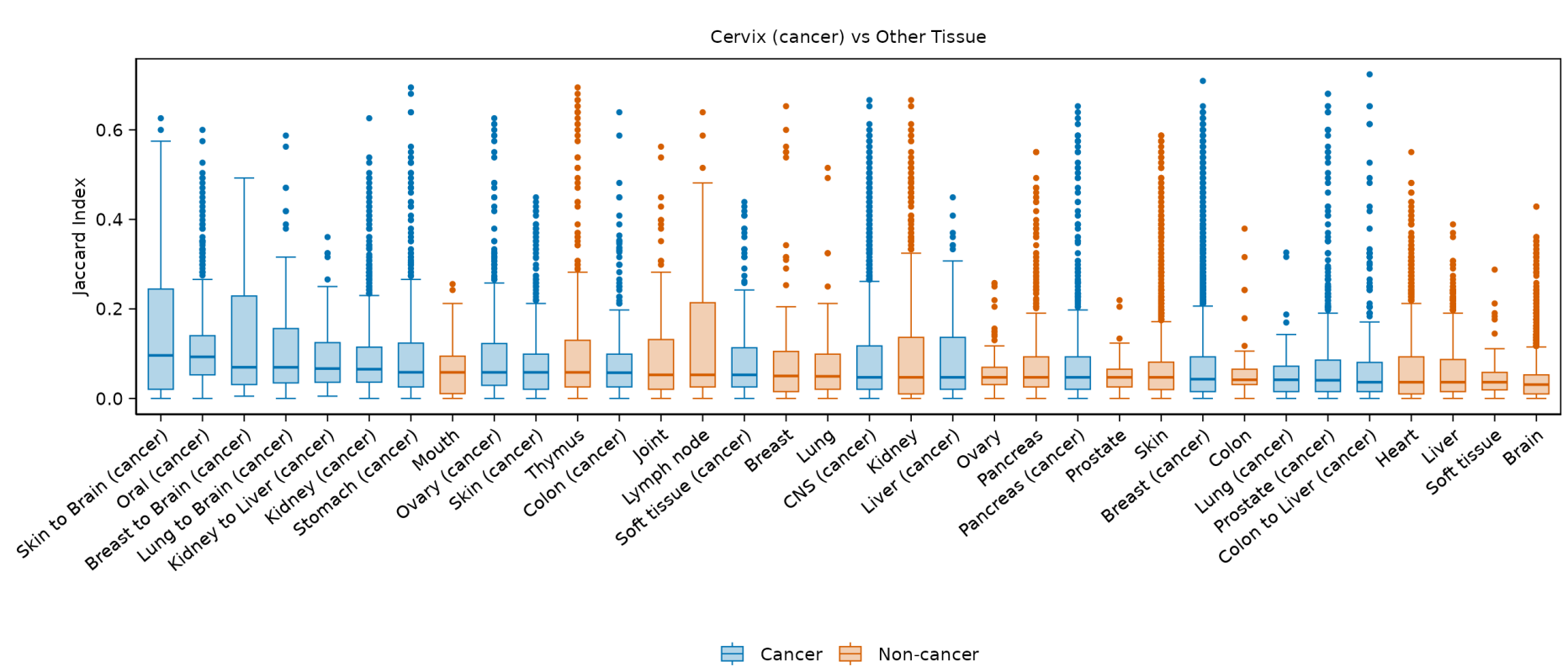

Supplementary Figure S19: Jaccard Index similarity between Cervix (cancer) vs Other Tissue

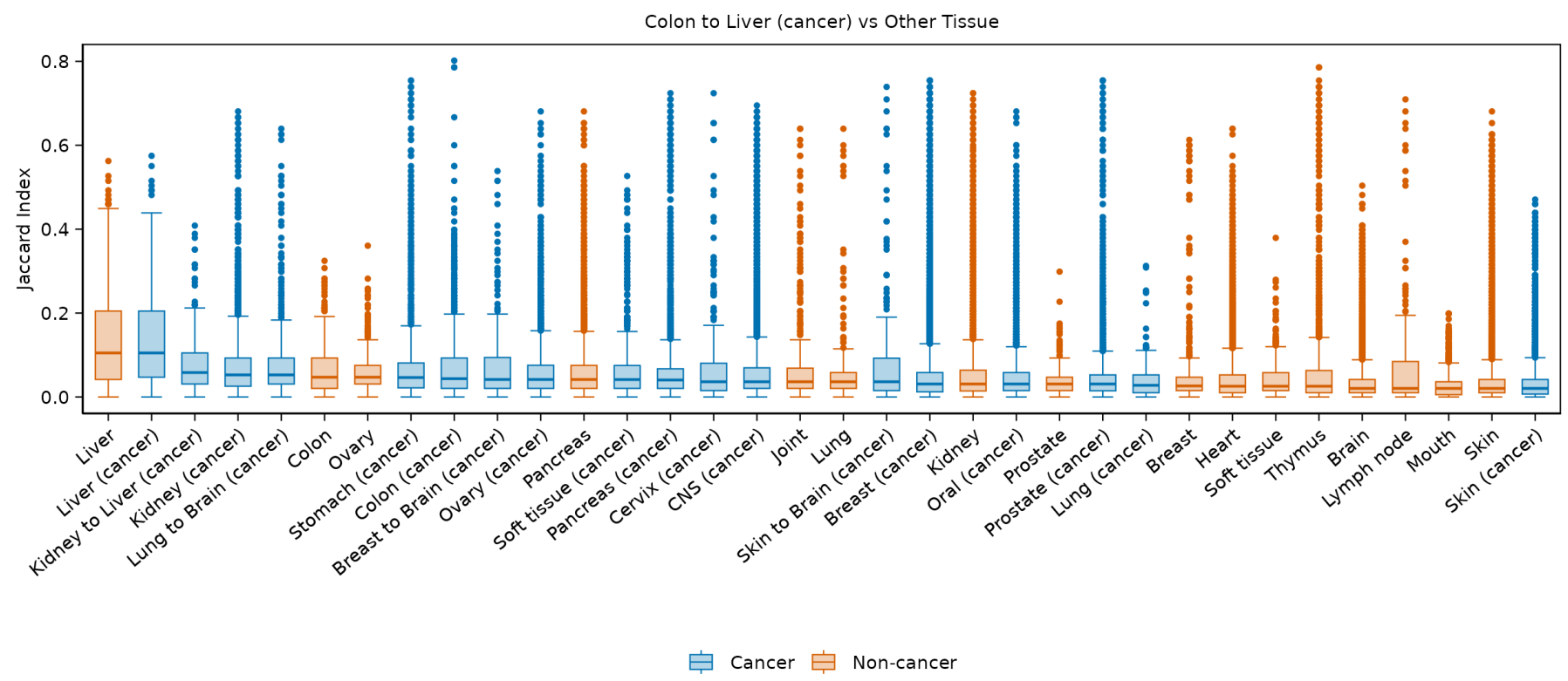

Supplementary Figure S20: Jaccard Index similarity between Colon to Liver (cancer) vs Other Tissue

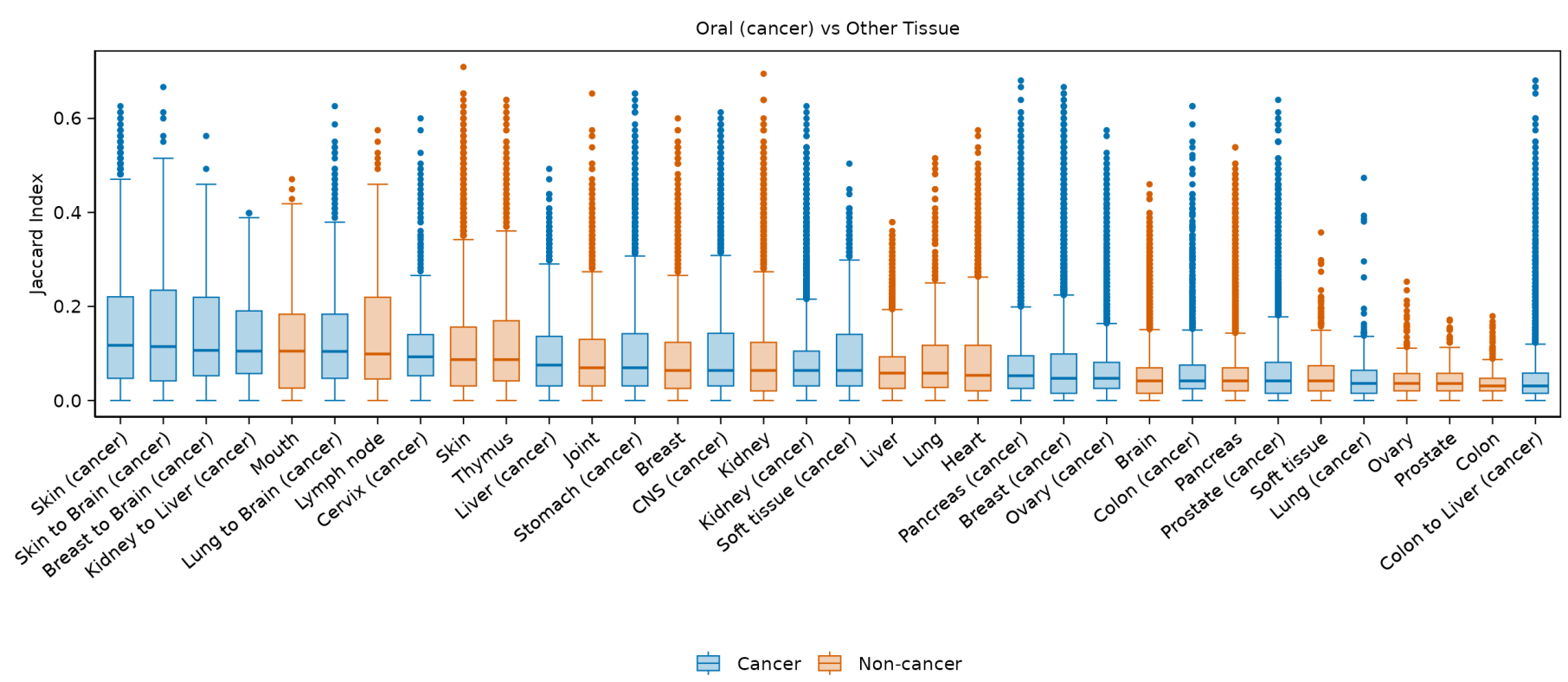

Supplementary Figure S21: Jaccard Index similarity between Oral (cancer) vs Other Tissue

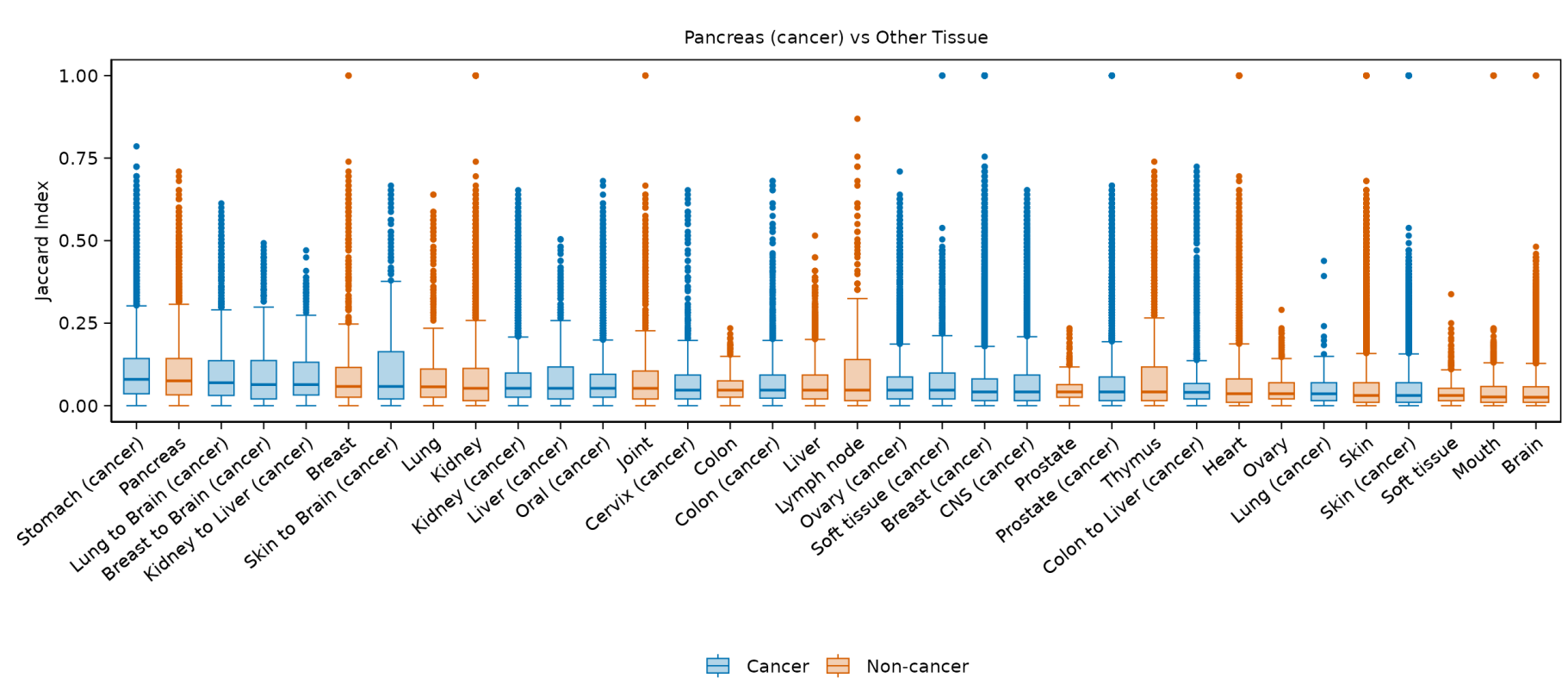

Supplementary Figure S22: Jaccard Index similarity between Pancreas (cancer) vs Other Tissue

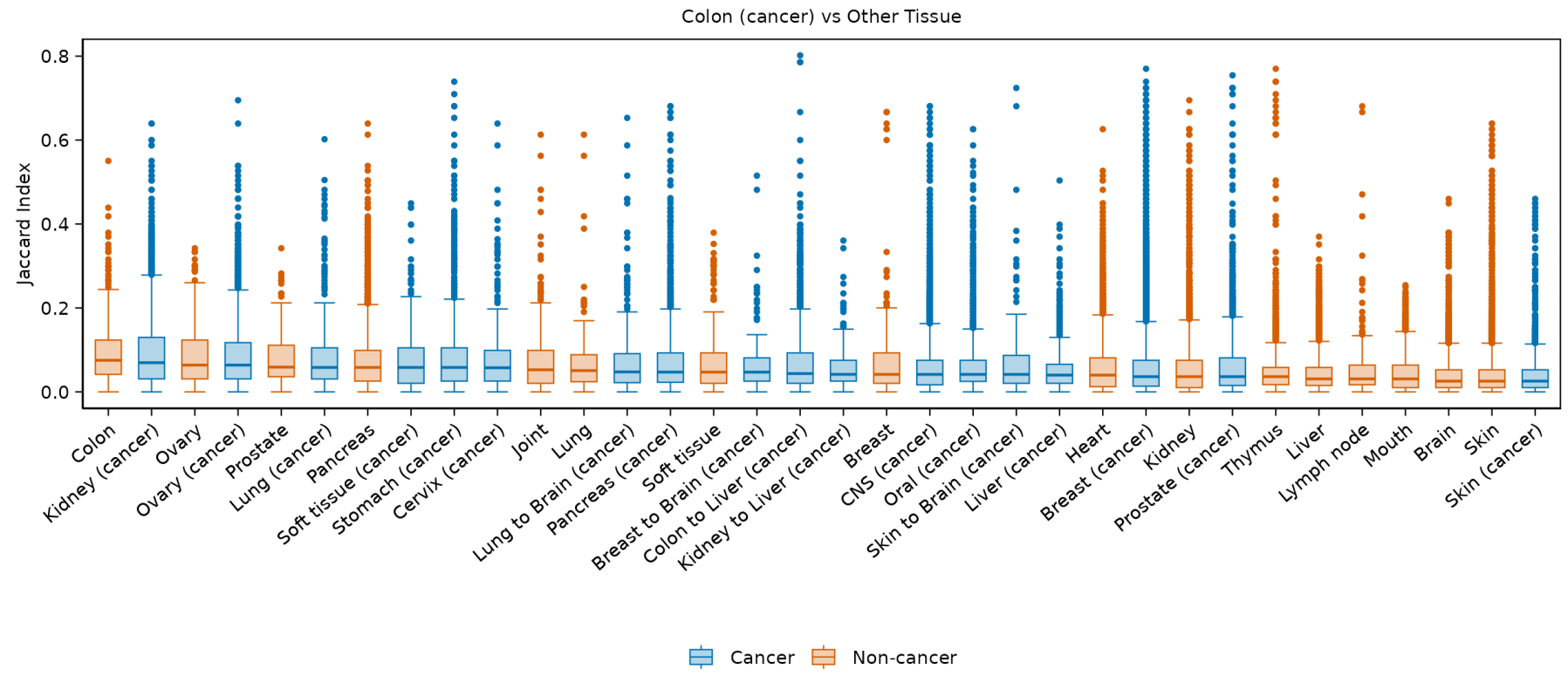

Supplementary Figure S23: Jaccard Index similarity between Colon (cancer) vs Other Tissue

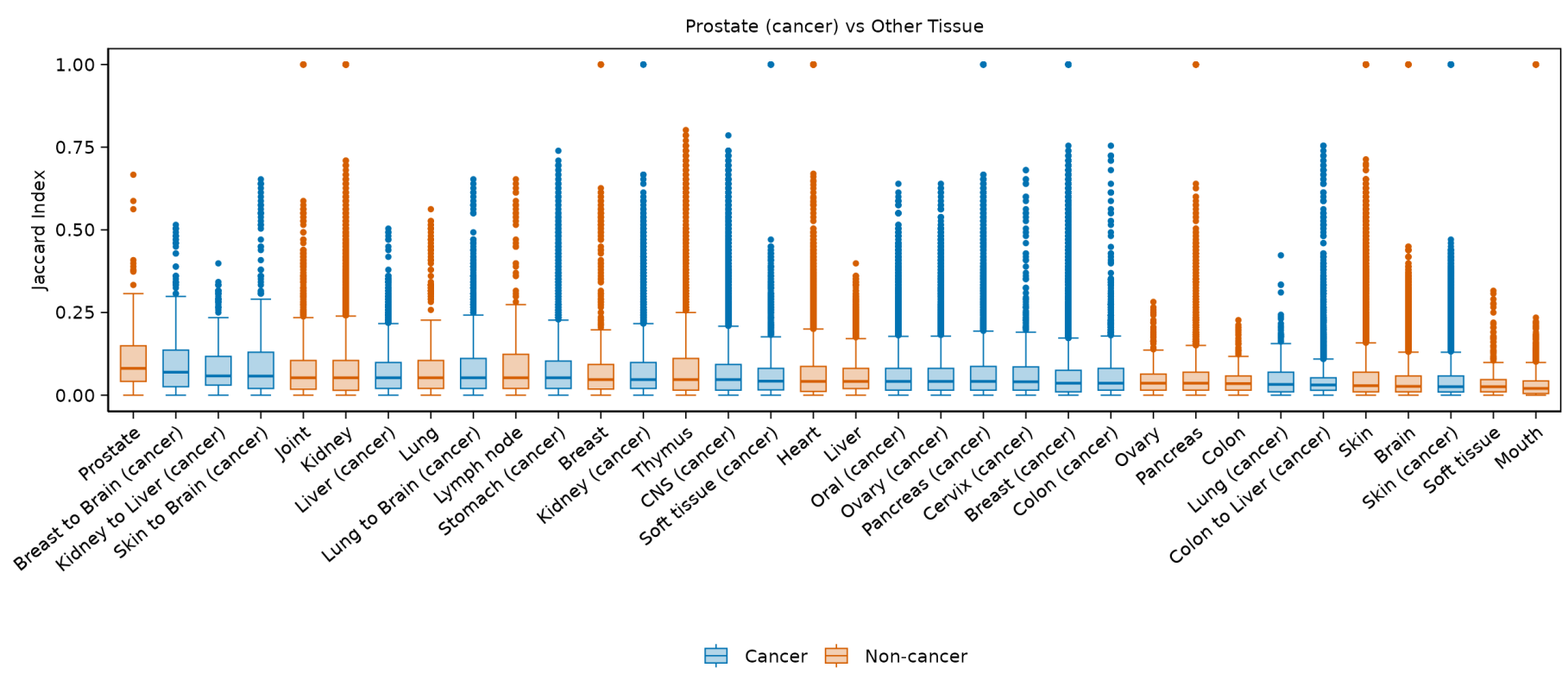

Supplementary Figure S24: Jaccard Index similarity between Prostate (cancer) vs Other Tissue

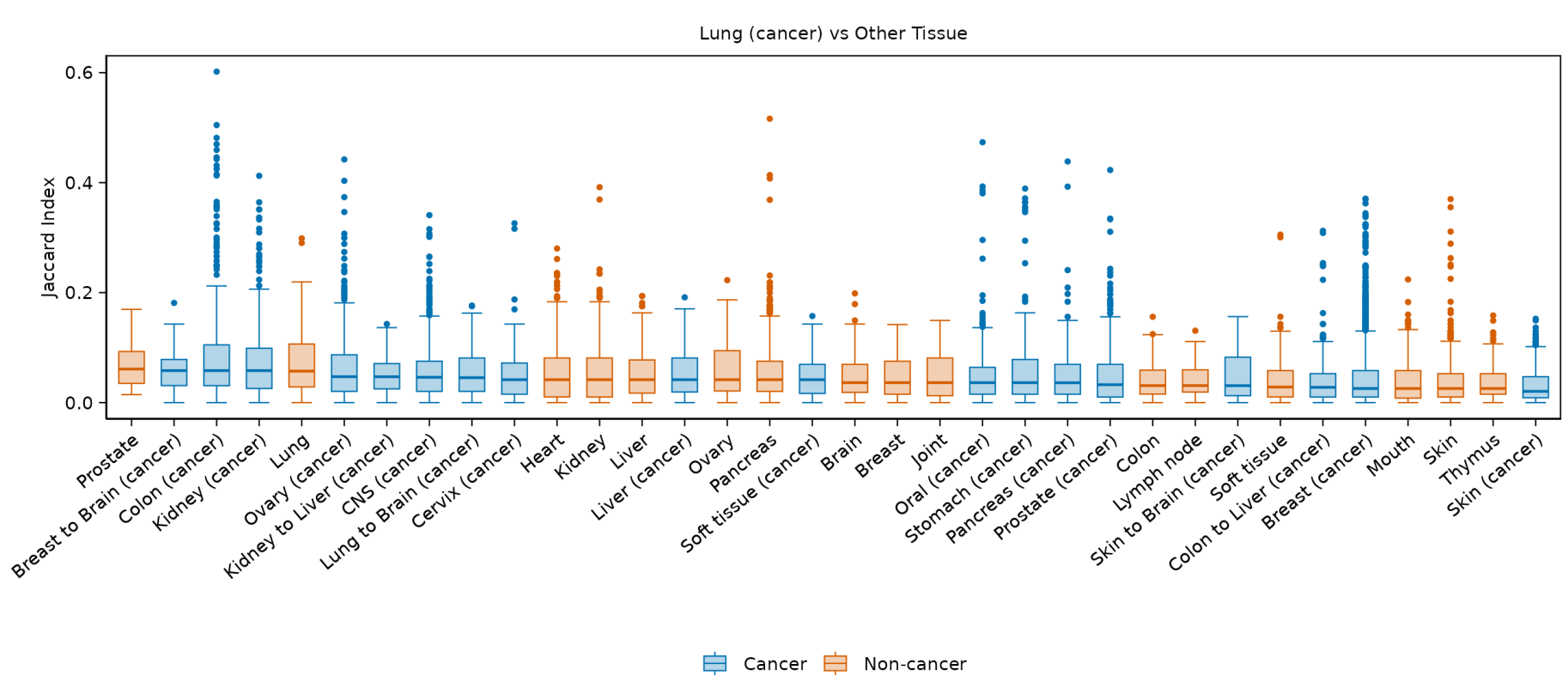

Supplementary Figure S25: Jaccard Index similarity between Lung (cancer) vs Other Tissue

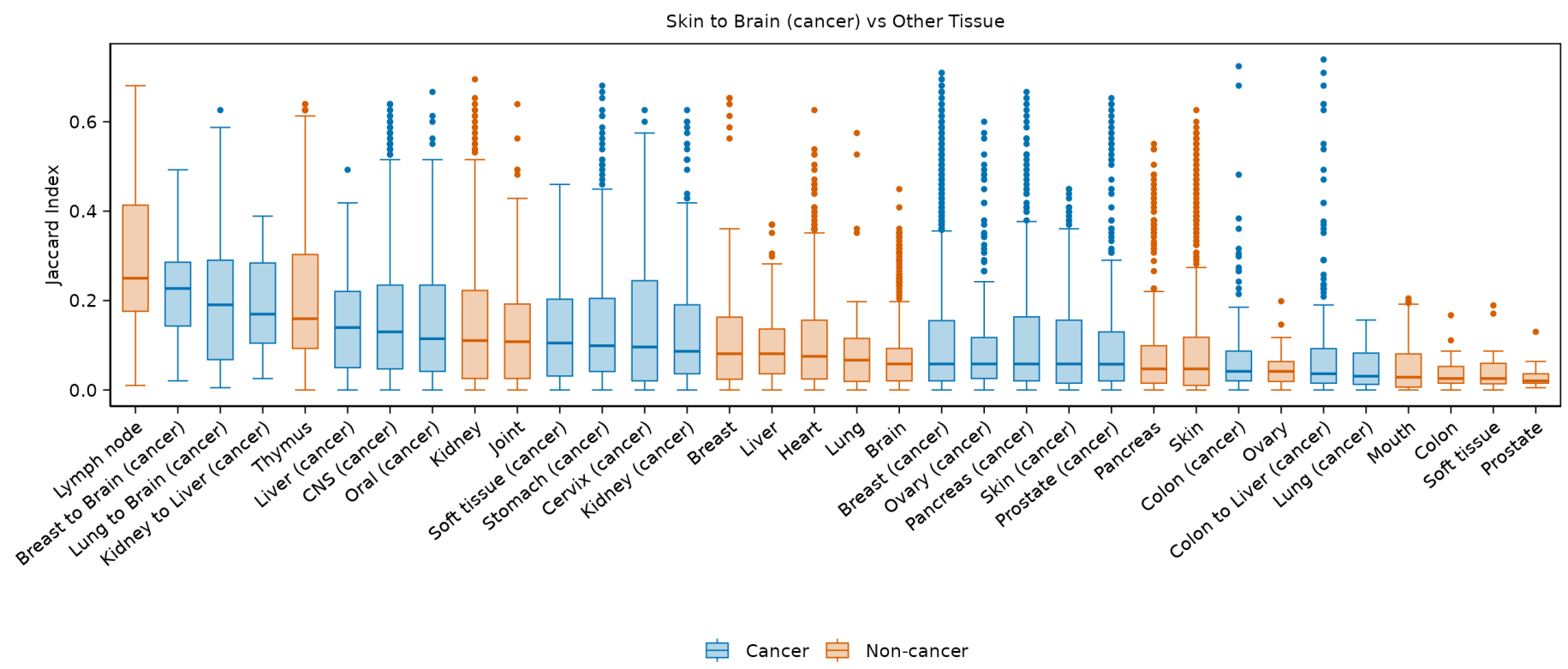

Supplementary Figure S26: Jaccard Index similarity between Skin to Brain (cancer) vs Other Tissue

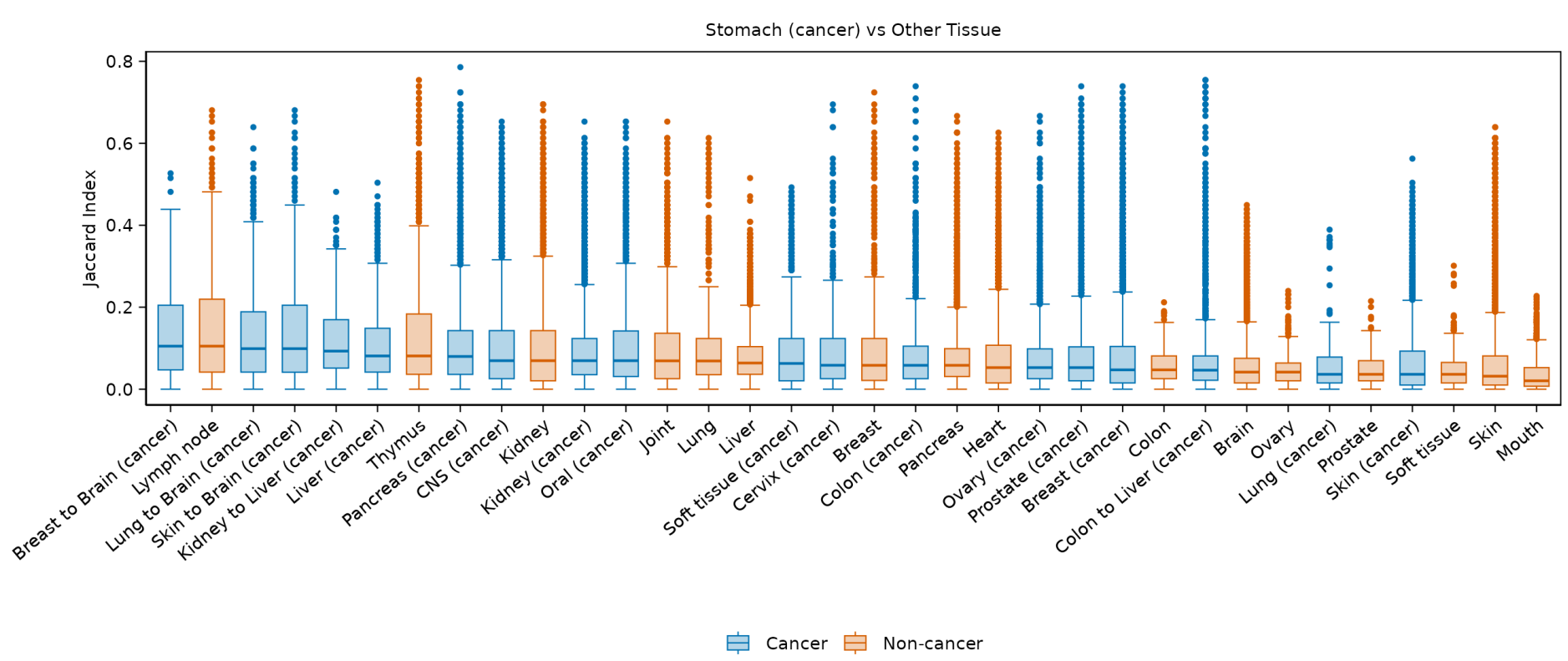

Supplementary Figure S27: Jaccard Index similarity between Stomach (cancer) vs Other Tissue

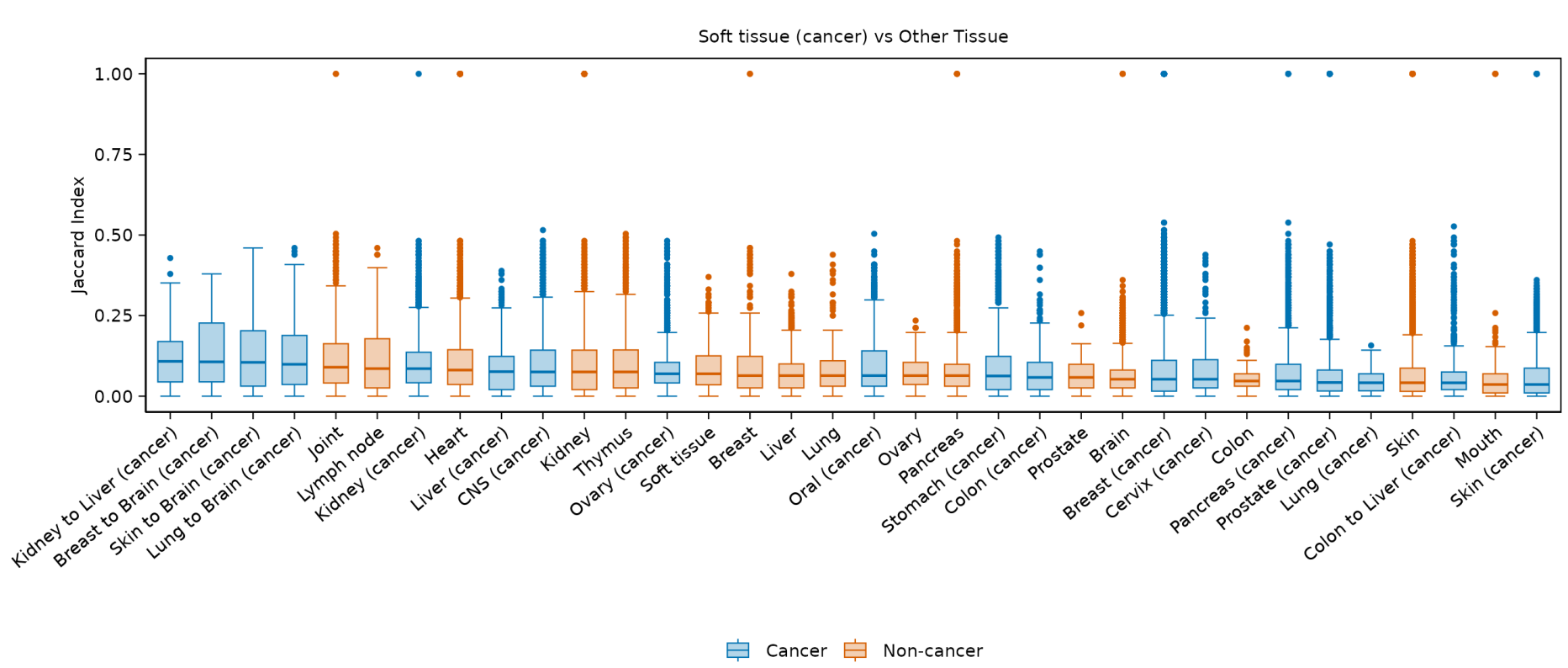

Supplementary Figure S28: Jaccard Index similarity between Soft tissue (cancer) vs Other Tissue

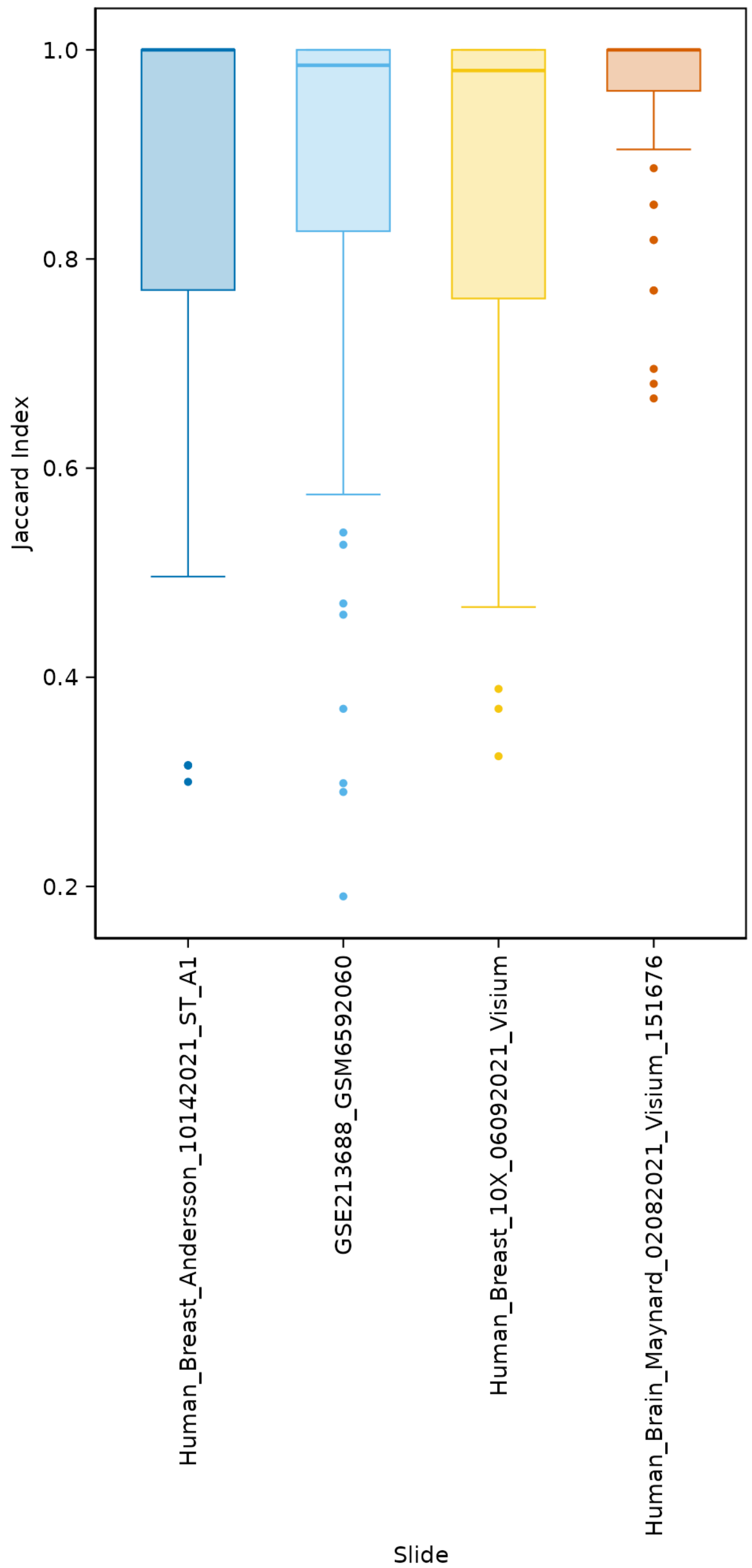

Supplementary Figure S29: Rotation robustness stratified by slides.

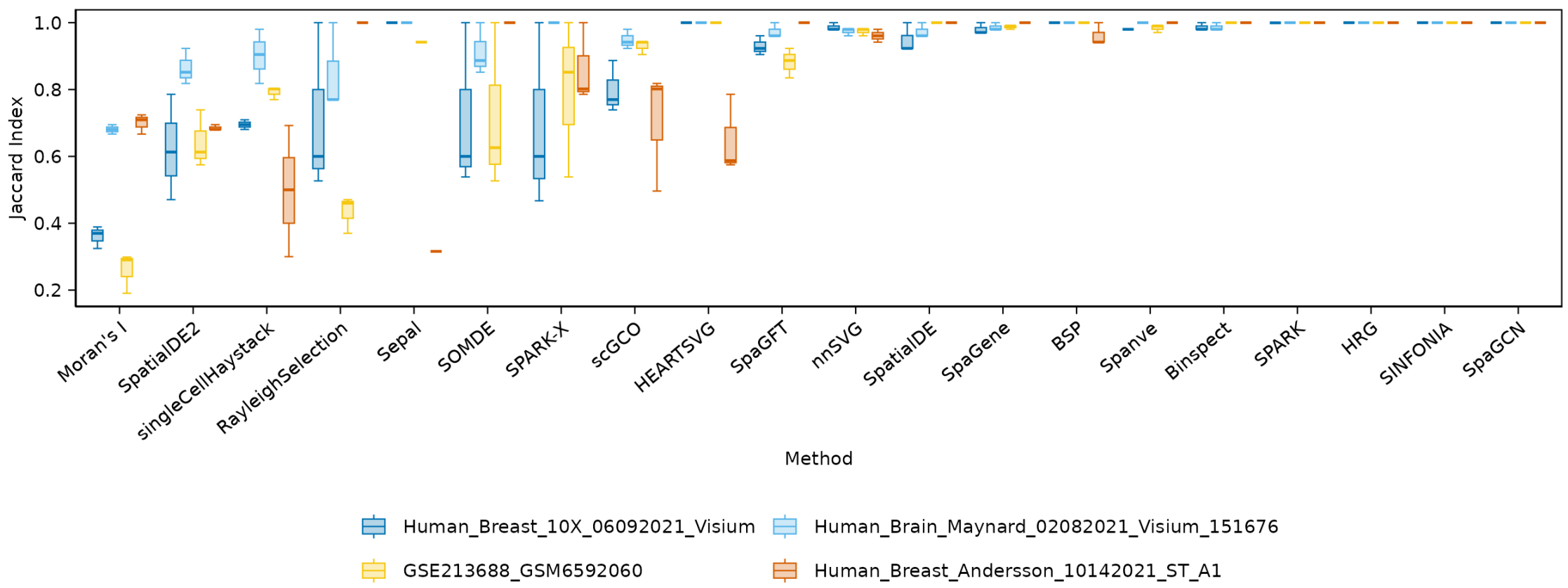

Supplementary Figure S30: Rotation robustness stratified by slides and methods.
